# A pyroptosis-associated signature plays a role in prognosis prediction in clear cell renal cell carcinoma

**DOI:** 10.1186/s12920-022-01339-0

**Published:** 2022-09-26

**Authors:** Zhiyuan Li, Zhinan Xia, Yipeng Yu, Licheng Cai, Wengang Jian, Tengda Wang, Wei Xue, Xingyuan Wang, Bowen Wang, Peng Zhang, Wenhao Yao, Cheng Zhang, Chunyang Wang

**Affiliations:** 1 Department of Urology, The First Affiliated Hospital of Harbin Medical University, Harbin, 150001 China; 2 Department of Urology, The Fourth Affiliated Hospital Zhejiang University School of Medicine, Yiwu City, 322000 China

**Keywords:** Clear cell renal cell carcinoma, Pyroptosis, Immune infiltration, Prognosis, Multiomics data, Functional enrichment analysis, Bioinformatics analysis

## Abstract

**Background:**

Approximately 90% of renal malignancies are RCCs (renal cell carcinomas), and the primary subtype in histology is ccRCC (clear cell RCC). In recent years, pyroptosis has been considered a kind of inflammation-related programmed cell death that participates in the invasion, metastasis, and proliferation of tumour cells, thereby influencing tumour prognosis. Nonetheless, the expression level of pyroptosis-associated genes in RCCs and their relationship with prognosis remain obscure.

**Results:**

In our research, 44 regulators of pyroptosis that were differentially expressed between normal kidney and ccRCC tissues were identified. ccRCC cases were categorized into 2 subgroups according to prognostic-related DEGs (differentially expressed genes), and there was a significant difference in OS (overall survival) between them. The prognostic value of pyroptosis-associated genes was assessed as a signature based on a cohort from TCGA (The Cancer Genome Atlas). Following Cox regression with DEGs and LASSO (least absolute shrinkage and selection operator), a 6-gene signature was established, and all ccRCC cases in the cohort from TCGA were categorized into an LR (low-risk) or HR (high-risk) group (*P* < 0.001). In combination with clinical features, risk scores were considered a predictive factor of OS in ccRCC. KEGG (Kyoto Encyclopedia of Genes and Genomes) and GO (Gene Ontology) analyses suggest increased immunity and enrichment of genes related to immunity in the HR group.

**Conclusions:**

Our findings indicate that genes related to pyroptosis have an important role in tumour immunity and may be used to predict the prognosis of ccRCC.

**Supplementary Information:**

The online version contains supplementary material available at 10.1186/s12920-022-01339-0.

## Introduction

RCCs (renal cell carcinomas) develop from renal tubular epithelial cells and account for approximately ninety percent of all kinds of renal malignancies. The main histological type is clear cell RCC (ccRCC) [1]. The incidence of RCCs in most countries has increased recently, with 400,000 new cases every year globally and over 175,000 deaths. The mortality rate and incidence of RCC rank 3rd among urological malignancies worldwide [2, 3]. Radical nephrectomy is the first-line treatment for regional renal cancer in the early stage, though distant metastasis or tumour recurrence occurs after surgery in over 20% of patients [4]. Moreover, RCC is characterized by tolerance to chemotherapy and traditional radiotherapy. Despite breakthrough advances in early diagnosis and comprehensive treatment of tumours in recent years, surgery remains the optimal treatment for ccRCC patients. Developing a specific prognostic strategy is of great importance to improve therapeutic effects.

Pyroptosis is a novel method of programmed cell death that is recognized as inflammation-related necrosis of cells [5] and is induced by a variety of stimuli, including heart attack, bacterial or virus infection, cancer, and stroke [6]. In addition to autophagy, apoptosis, and ferroptosis, this kind of cell death has attracted much attention recently.

The characteristics of pyroptosis are cell swelling, large bubbles moving from the membrane, and lysis [7]. Caspase-1 [ICE for IL (interleukin)-converting enzyme], a member of the inflammatory caspase family, was the first kind of caspase discovered to be involved in facilitating pro-IL-1b to form mature IL-1b [8, 9]. Caspase-1-dependent plasma membrane pores require gradients of cellular ions, contributing to an increase in osmotic pressure, which results in cell swelling and water influx [10]. Caspase-1 dependence, which regulates cell lysis, is a specific characteristic of pyroptosis but not apoptosis [11–13]. The gasdermin family is a major executor in pyroptosis and contains pejvakin (PJVK or DFNB59) and gasdermin-A to gasdermin-E [14]. Inflammasomes participate in activation of Caspase-1, resulting in GSDMD (gasdermin D) cleavage and the maturation and secretion of proinflammatory factors, including IL-1B and IL-18 [15]. In addition to GSDMD, cleavage of other kinds of gasdermin proteins also induces the formation of pores on the plasma membrane. In particular, Caspase-3 participates in cleavage of GSDME (gasdermin E) to induce pyroptosis [16, 17].

According to previous studies, pyroptosis plays a pivotal role in the development of malignancies and antitumor activities. For instance, recent research identified a novel gene signature related to pyroptosis that may be used in the prognosis of skin cutaneous melanoma [18]. Nonetheless, the specific effects of pyroptosis remain to be explored in ccRCC. Hence, this systematic study was conducted to explore expression differences of genes related to pyroptosis in ccRCC and normal tissues, and a new PRG (pyroptosis-related gene) prognostic risk signature in ccRCC was established to predict survival. Furthermore, phenotypes related to prognosis and relationships between the immune microenvironment of tumours and pyroptosis were investigated.

## Materials and methods

### Collection of TCGA data

Transcriptome RNA sequencing data and clinical data for 611 ccRCC cases (72 normal samples and 539 tumour samples) were downloaded (website: https://portal.gdc.cancer.gov/; TCGA database). Samples without complete information were excluded.

### Identification of DEGs related to pyroptosis

A total of 52 genes associated with pyroptosis were extracted from prior reviews [19–26], as shown in Additional file 2: Table S1. The “limma” package was used to find DEGs associated with pyroptosis between tumour and normal tissues, and 44 DEGs were identified according to *P* < 0.05. Version 11.0 STRING (Search Tool for the Retrieval of Interacting Genes; website: https://string-db.org/) was used to construct a PPI network. The R programming language and “pheatmap” package were utilized to acquire heatmaps of the DEGs. The “igraph” and “reshape2” R packages were applied to evaluate relationships between selected DEGs (cut-off = 0.2).

### Consensus clustering

To categorize ccRCC according to consensus clustering, the “limma”, “survival” and “ConsensusClusterPlus” R packages were applied. Prognostic PRGs in diverse subgroups were screened with the "limma" R package (LogFC = 1, FDR = 0.05). Relationships between clinical characteristics (OS, overall survival) and clusters were evaluated using the R package “survival” and the chi-square test. To present the results, Kaplan‒Meier (KM) curves and heatmaps were generated using the R packages “pheatmap”, “survival”, and “survminer”.

### Development of a ccRCC prognostic model based on PRGs

Univariate Cox regression analysis was executed by using the “survival” R package, and the significance filter was set to 0.05. Cox regression analysis (least absolute shrinkage and selection operator, LASSO) was conducted with the R package “glmnet” to construct a prognosis model using candidate genes. The minimum parameters were used to determine the penalty parameter (λ). The following equation was used to calculate the RS (risk score): the RS equals e^Σi(Coefi·Expi)^, where Expi denotes the expression level of each retained gene and Coefi the coefficient. Principal component analysis (PCA) was performed by applying the R package “Rtsne” according to the risk score. The R packages “survival” and “survminer” were applied for KM analyses. The R package “survivalROC” was employed for 1-, 3- and 5-year ROC analyses in different populations.

### Analysis of the prognostic value of the RS

Clinical features (age, grade, sex, and T and M classification) of the patients in the cohort from TCGA were obtained. The RS and these extracted variables were analysed together in the regression model, with multivariable and univariate Cox regression models used.

### Weighted gene coexpression network analysis (WGCNA)

First, we performed a screen for DEGs among 539 KIRC samples in the cohort from TCGA by using R software and the "limma" package. By setting screening conditions of |log2FC|> 1 and *P* < 0.05, 4484 DEGs were identified. We subsequently tested the suitability of these genes and used the R package "WGCNA" to construct gene coexpression networks. An adjacency matrix was built to calculate the strength of association between nodes by the following formula: a_ij_ =|S_ij_|^β^ (a_ij_: adjacency matrix between gene i and gene j, S_ij_: similarity matrix which was achieved by Pearson correlation of all gene pairs, β: softpower value). In this study, the soft threshold was 2, and the scale-free exponent was 0.9. Then, we transformed the adjacency matrix into a topological overlap matrix (TOM), which is a method to quantitatively describe the similarity of nodes by comparing the weighted correlation between two nodes and other nodes. Hierarchical clustering was performed to distinguish differential modules, with each containing at least 50 genes (minModuleSize = 50). Finally, we merge similar modules by computing the correlation between modules (abline = 0.25).

### Analysis of gene set enrichment

The cohort patients with ccRCC from TCGA were assigned to 2 subgroups based on the median RS. DEGs between the HR (high-risk) group and the LR (low-risk) group were screened based on the criteria FDR < 0.01 and |log2FC|≥ 1.5. KEGG and GO analyses of DEGs were conducted using the “clusterProfiler” package. ssGSEA was carried out by using the “pheatmap package” and “gsva” packages to assess activity of the pathways linked to immune responses after determination of the scores of IIC (immune cell infiltration). Gene set enrichment analysis (GSEA) was used in TCGA-KIRC to identify the potential regulatory mechanism for six biomarkers between the high- and low-expression subgroups via GSEA software (GSEA version 4.1.0), and the R package named “ggplot2” was used to prepare Additional file 1: Fig. S4. The c2.cp.kegg.v7.5. symbols.gmt gene set from the KEGG database was selected as the reference gene set. Biological processes with a normalized *p* < 0.05 and a false discovery rate (FDR) *q* < 0.05 were considered statistically significant.

### TIMER database analysis

Comprehensive analysis was conducted using the TIMER database (website: https://cistrome.shinyapps.io/timer), and IIC in over ten thousand tumours of thirty-two types of cancers was visualized [27]. Six subsets of tumour-infiltrating immune cells (macrophages, dendritic cells, CD8 T cells, neutrophils, CD4 T cells, and B cells) are included in TIMER. A new statistical approach was used to assess abundance of the 6 types of infiltrating immune cells in the tumour microenvironment. The genomic, immunological, and clinical features of the tumour were comprehensively investigated by using the TIMER database. For each hub gene in the risk score model, the SCNA (somatic copy number alteration) module of the TIMER tool was used to compare infiltration between ccRCC samples, including different SCNAs, such as high amplification, diploid/normal, deep deletion, arm-level deletion, and arm-level gain [28]. Additionally, the infiltration level in ccRCC samples was collected using the TIMER database to determine relationships with the RS system and six hub genes.

### CIBERSORT

The proportion of 22 tumour infiltrating immune cells in each sample was determined by using "CIBERSORT" (R package). CIBERSORT predicts the proportion of 22 immune cells in each tissue by analysing the relative expression levels of 547 genes in a single tissue sample based on gene expression profiling [29]. Normalized gene expression profiles of ccRCC were transformed into proportions of 22 IICs. Relative expression of the 22 IICs in each sample was then determined. Significant results (*P* < 0.05) were selected for subsequent analysis. Correlation analysis and scatter plot drawing were conducted in the "limma" and "ggplot2" software packages.

### Statistics

R software (version 4.1.0) and the packages mentioned above were used for statistical analyses. The log-rank test and K-M method were employed for survival analyses. The significance of prognostic factors was evaluated by using multivariate and univariate Cox regression analyses. The Kruskal–Wallis test and Wilcoxon rank-sum test were applied for subgroup differential analyses. All statistical tests were two-sided. *P* < 0.05 was regarded as statistically significant.

## Results

### Identification of differentially expressed PRGs between normal and tumour tissues

**Fig. 1:**
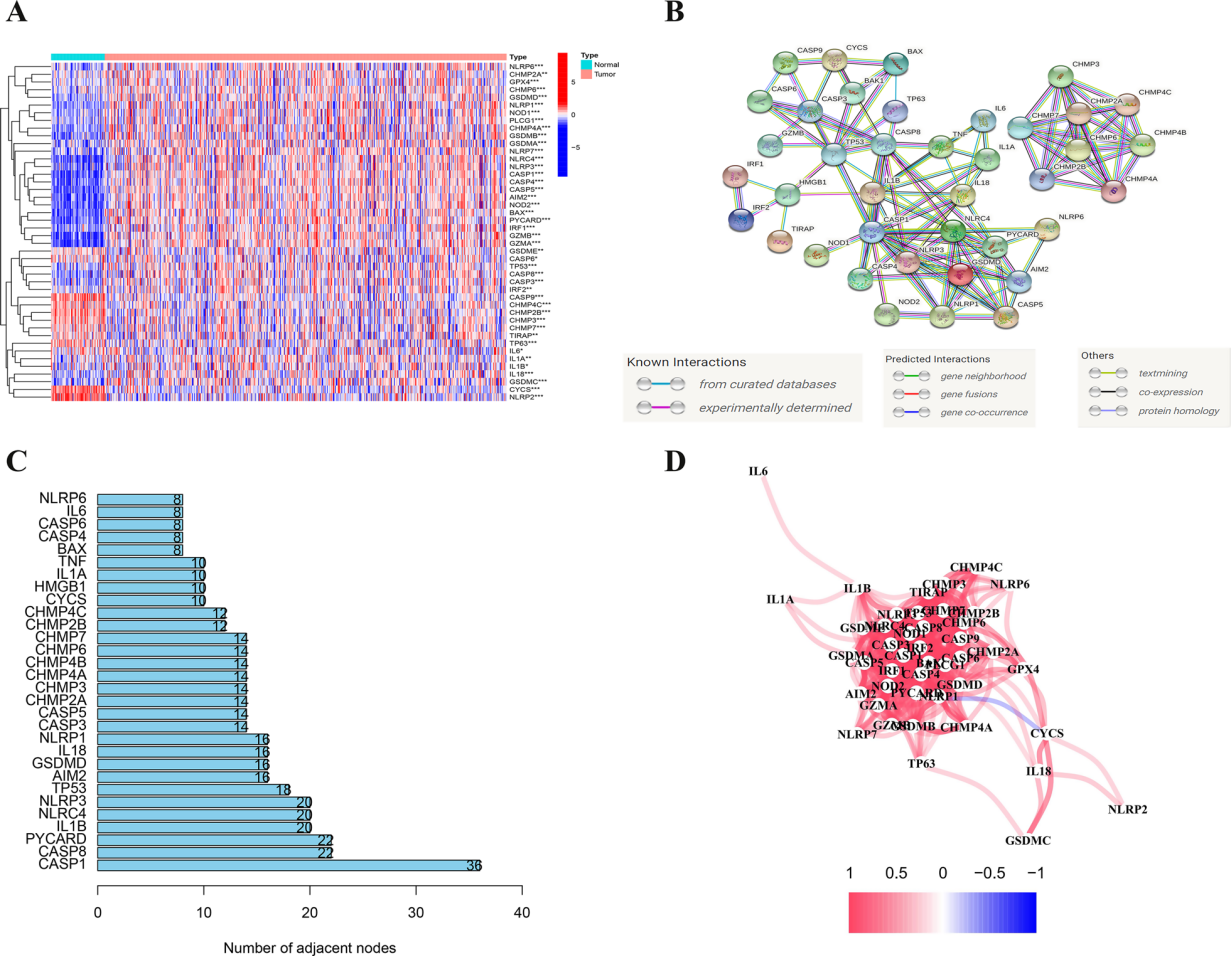
Expressions and interactions of 44 pyroptosis-linked genes. **A** Heatmap (blue: low level of expression; red: high level of expression) of genes linked to pyroptosis between tumour (T, brilliant red) and normal (N, brilliant blue) tissues. ***P < 0.001, **P < 0.01, *P < 0.05. **B** Interaction between genes linked to pyroptosis indicated by the PPI network (the interaction score was 0.9). **C** Hub genes ranked by node number. **D** The correlation network of genes related to pyroptosis (blue colour, negative; red colour, positive), whereby the depth of colour represents the relative strength

Expression levels of 52 genes related to pyroptosis in the database TCGA (The Cancer Genome Atlas) were compared between 539 tumours and 72 normal tissues. Ultimately, 44 DEGs were identified. Thirteen genes (NLRP2, TP63, CYCS, CASP9, IL1A, CHMP2B, CHMP4C, CHMP3, IL1B, CHMP7, TIRAP, CASP6, and GSDME) were downregulated and 31 other genes (CHMP2A, IRF2, CHMP6, TP53, GPX4, CASP3, PLCG1, NOD1, GSDMD, CASP8, CHMP4A, IL18, IL6, IRF1, NLRP1, CASP4, BAX, NLRP3, NLRP6, GSDMA, CASP1, GSDMB, NLRC4, PYCARD, NLRP7, GSDMC, NOD2, GZMB, CASP5, AIM2, GZMA) upregulated in tumour tissue samples. Heatmaps were used to visualize RNA expression of these genes (Fig. 1A, blue: low level of expression; red: high level of expression).

To further investigate interactions between these PRGs, PPI (protein‒protein interaction) analysis was conducted by applying the STRING platform. The results are illustrated in Fig. 1B. The minimum interaction score required for PPI analysis was 0.9 (the highest confidence). The top 30 hub genes according to the number of nodes are listed in Fig. 1C. Moreover, except for CHMP4B and HMGB1, DEGs between normal tissues and tumour tissues are shown. The network of correlations including all genes linked to pyroptosis is shown in Fig. 1D (blue colour, negative; red colour, positive).

### Tumour categorization according to DEGs linked to pyroptosis

**Fig. 2:**
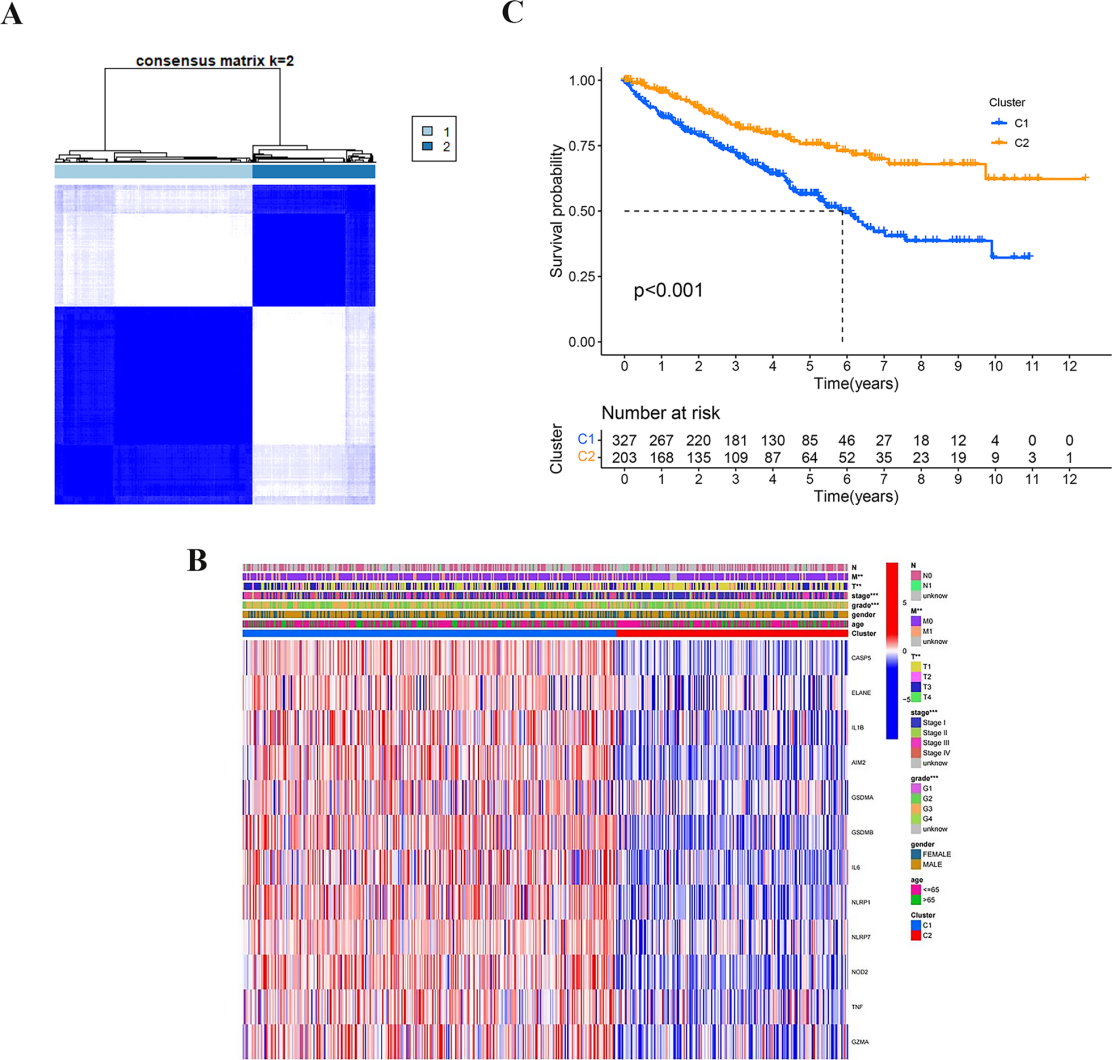
Tumour categorization according to PRGs related to prognosis. **A** Total of 539 ccRCC patients were assigned into 2 clusters based on the CCA matrix (k = 2; High expression level cluster of prognostic PRGs, C1; Low expression level cluster of prognostic PRGs; C2). **B** The clinicopathologic features and heatmap of the 2 clusters categorized by PRGs (T, N, and M classification included lymph node metastasis, tumour size, and distant metastasis) (P values: *P < 0.05; **P < 0.01; ***P < 0.001). **C** Kaplan–Meier OS curves for the 2 clusters

To investigate the relationship of ccRCC subtypes with the 52 PRG levels, we screened prognostic PRGs by applying univariate Cox analysis and clustered the samples by the PAM (partitioning around medoid) computational method based on their expression levels. CCA (consensus clustering analysis) was performed by using data for 539 ccRCC (Additional file 6: Table S5) samples in TCGA. Intragroup correlation peaked when the clustering variable (k) increased raised from two to ten; intergroup correlation was lowest when k = 2, indicating that the 539 ccRCC patients could be well divided into two groups according to 52 PRGs, as shown in Fig. 2A. The clinical characteristics and gene expression profile are presented in a heatmap, and differences between patient characteristics between the two clusters are shown in Additional file 7: Table S6. After comparing Cluster 1 with Cluster 2, we observed significant differences in clinical stage (stage I–IV), distant metastasis (M0-1), Fuhrman grade (G1–G4), and tumour size (T1–T3); conversely, differences in the number of LNM (lymph node metastases), sex and age were not statistically significant (Fig. 2B). We also compared the OS of the 2 clusters, which is presented in Fig. 2C. OS was significantly poorer in Cluster 1 than in Cluster 2 (*P* < 0.001, Fig. 2C).

### Prognostic value of the PRG expression signature in ccRCC

**Fig. 3:**
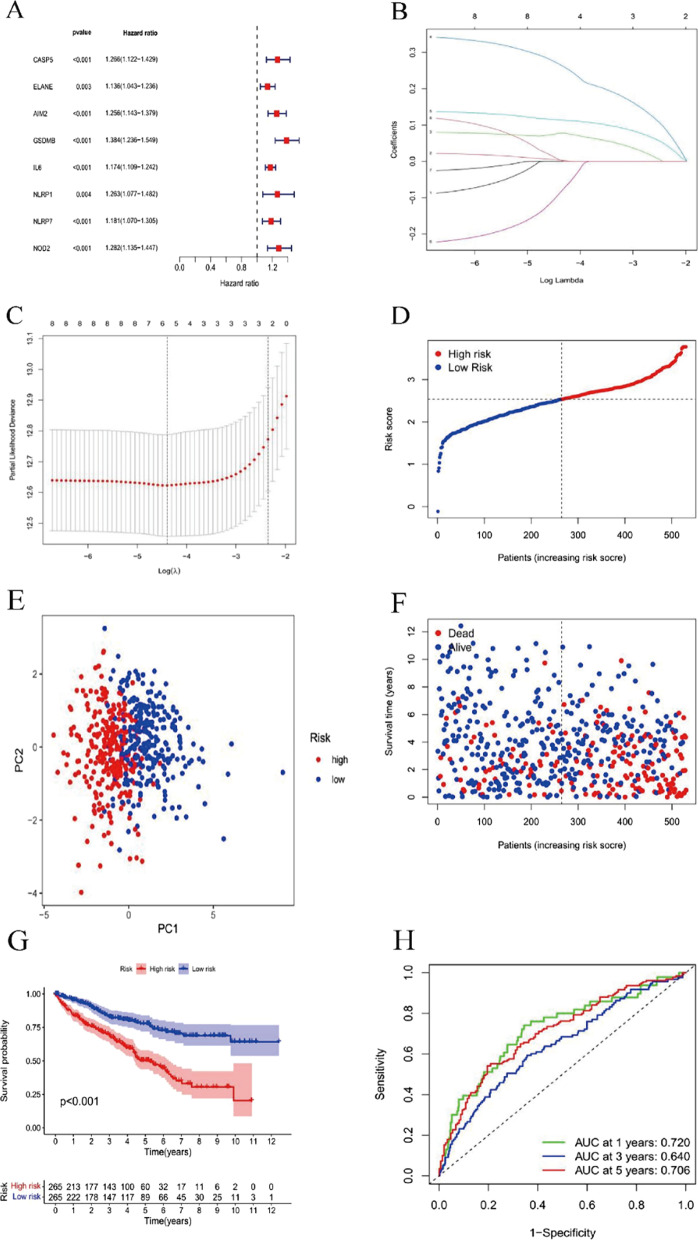
Risk signatures were constructed using the cohort from TCGA. **A** ccRCC was analysed by univariate Cox regression for every DEG linked to pyroptosis and 8 genes with P less than 0.05. **B** Six genes linked to OS were analysed by LASSO regression. **C** The selection of parameters was cross-validated. **D** Case distribution according to the RS. **E** Principal component analysis for ccRCCs according to the RS. **F** Survival of every patient (the right side of the dark line, HR cases; the left side, LR cases). **G** KM curves for ccRCC cases in the LR group and HR group. **H** Predictive efficiency of the RS indicated by ROC curves

In total, 530 ccRCC samples were matched with corresponding cases with complete information about survival. We used univariate Cox regression analysis to initially screen for survival- and pyroptosis-related DEGs, and 8 genes with *P* < 0.05 were used for analysis (Fig. 3A). To screen candidate genes for construction of the model of prognosis, Cox regression analysis (LASSO) was performed. Based on the optimal λ value, 6 genes (Fig. 3B, C) and their coefficients (Table 1) were eventually retained, and 530 patients were equally assigned into high- and low-risk subgroups according to the median score (Fig. 3D; Additional file 7: Table S6). As shown in Fig. 3E, PCA demonstrated that subjects with alternative risks could be divided into 2 clusters. The death rate was higher and survival time shorter in the high-risk group than in the low-risk group (as shown in Fig. 3F). In addition, we assessed the distribution of risk scores between the clusters, with significantly more high-risk patients in Cluster 1 than in Cluster 2 (Additional file 1: Fig. S1A, B). As depicted in Fig. 3G, there was a significant difference in ccRCC, and lifespan was shorter in the high-risk subgroup (*P* < 0.001, HR = 0.374, 95% CI 0.277–0.504). As continuous variables, a certain correlation between the RS and OS remained (Additional file 1: Fig. S2). The specificity and sensitivity of the prognostic model were assessed by applying a ROC (receiver operating characteristic) curve, and the AUC (area under the ROC curve) was 0.706, 0.640, and 0.720 for 5-year, 3-year, and 1-year survival, respectively (Fig. 3H). As illustrated in Additional file 1: Fig. S3, we estimated the prognostic value in different populations, including females, males, age ≥ 60, age < 60, stage 1–2, stage 3–4, grade 1–2, and grade 3–4. Our results indicate that the PRG expression signature is valuable for predicting prognosis in ccRCC.

**Table 1:** Construction of a pyroptosis-related signature in ccRCC

Gene name	Coefficients	HR (95% CI)	*P* value
ELANE	0.003234381	1.136 (1.043–1.236)	0.003221789
AIM2	0.077194693	1.256 (1.143–1.379)	2.00E-06
GSDMB	0.267608183	1.384 (1.236–1.549)	1.82E-08
IL6	0.113880538	1.174 (1.109–1.242)	3.33E-08
NLRP1	− 0.089187177	1.263 (1.077–1.482)	0.00404868
NOD2	0.004289527	1.282 (1.135–1.447)	6.28E-05

HR, hazard ratio; CI, confidence interval

### Prognostic value of the risk model in ccRCC

**Fig. 4:**
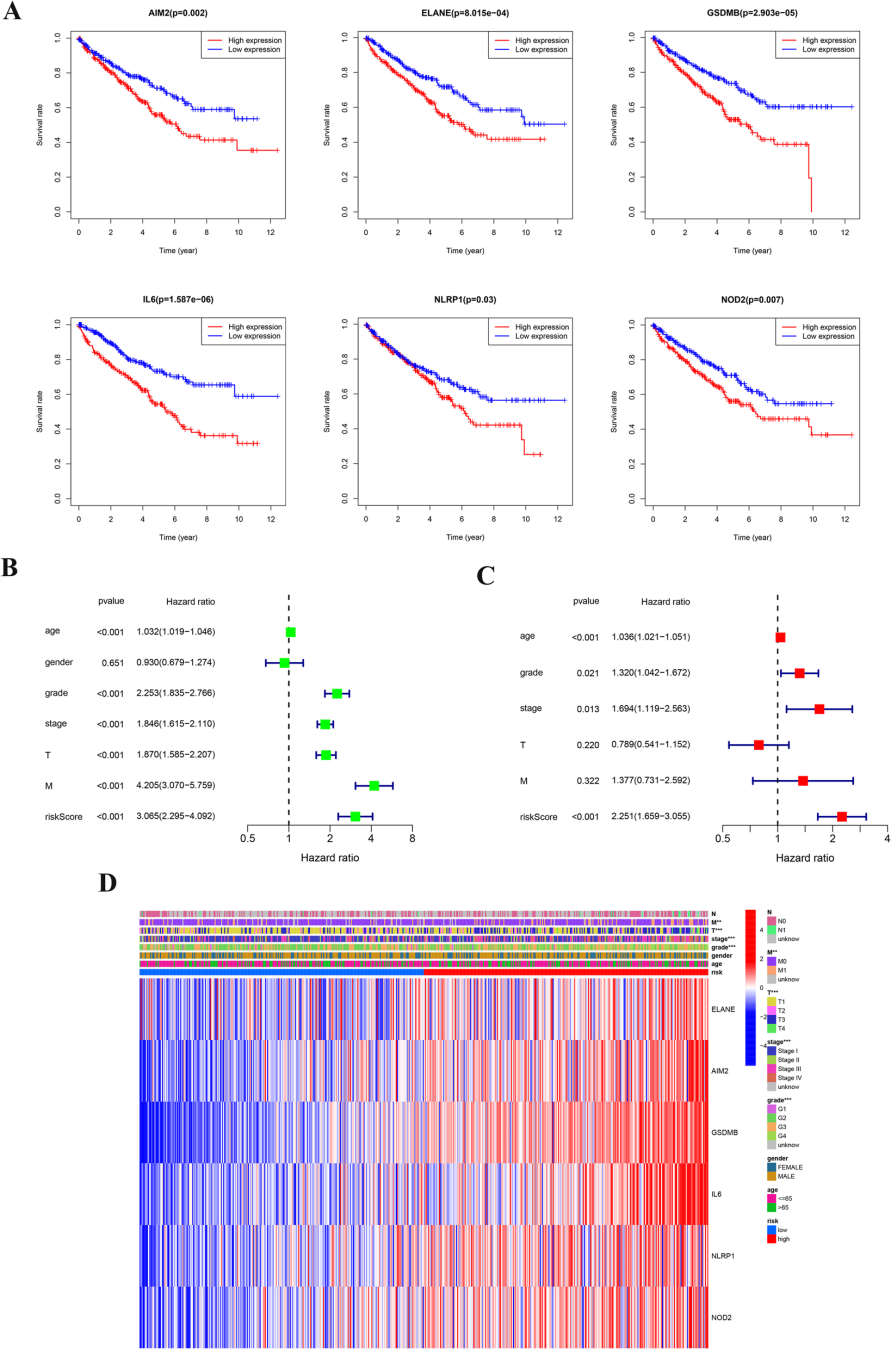
The RS was analysed by Cox regression. **A** Kaplan–Meier analysis of the six hub genes linked to pyroptosis (ELANE, AIM2, GSDMB, IL6, NLRP1, and NOD2) in TCGA. **B** Results of univariate analysis of the cohort from TCGA. **C** Results of multivariate analysis the cohort from TCGA. **D** Heatmap (blue colour, downregulated expression; red colour, upregulated expression) of relationships of clinical characteristics with risk groups (***P < 0.001, **P < 0.01, *P < 0.05)

To identify the prognostic value of the risk model, we conducted a Kaplan‒Meier analysis to confirm whether the genes involved in the construction of the risk model are associated with the prognosis of ccRCC. As indicated in Fig. 4A, higher levels of ELANE, AIM2, GSDMB, IL6, NLRP1, and NOD2 correlated positively with poor prognosis. We then analysed whether the RS of the gene signature model is a factor predicting prognosis, and the results suggested that the RS is a predictive factor for prognosis in the cohort from TCGA (HR = 3.065, 95% CI 2.295–4.092, Fig. 4B). According to the results of multivariate analysis, after adjustment for confounding factors, the RS was found to be a predictive factor for the prognosis of patients with ccRCC in TCGA (HR = 2.251, 95% CI 1.659–3.055, Fig. 4C), and a heatmap of clinical characteristics was generated (Fig. 4D), with a significant difference in tumour grade and stage between subgroups. These findings suggest that the prognostic model based on PRGs is robust and independent in the prediction of ccRCC prognosis.

### Identifying prognostic model-related BPs (biological processes)

**Fig. 5:**
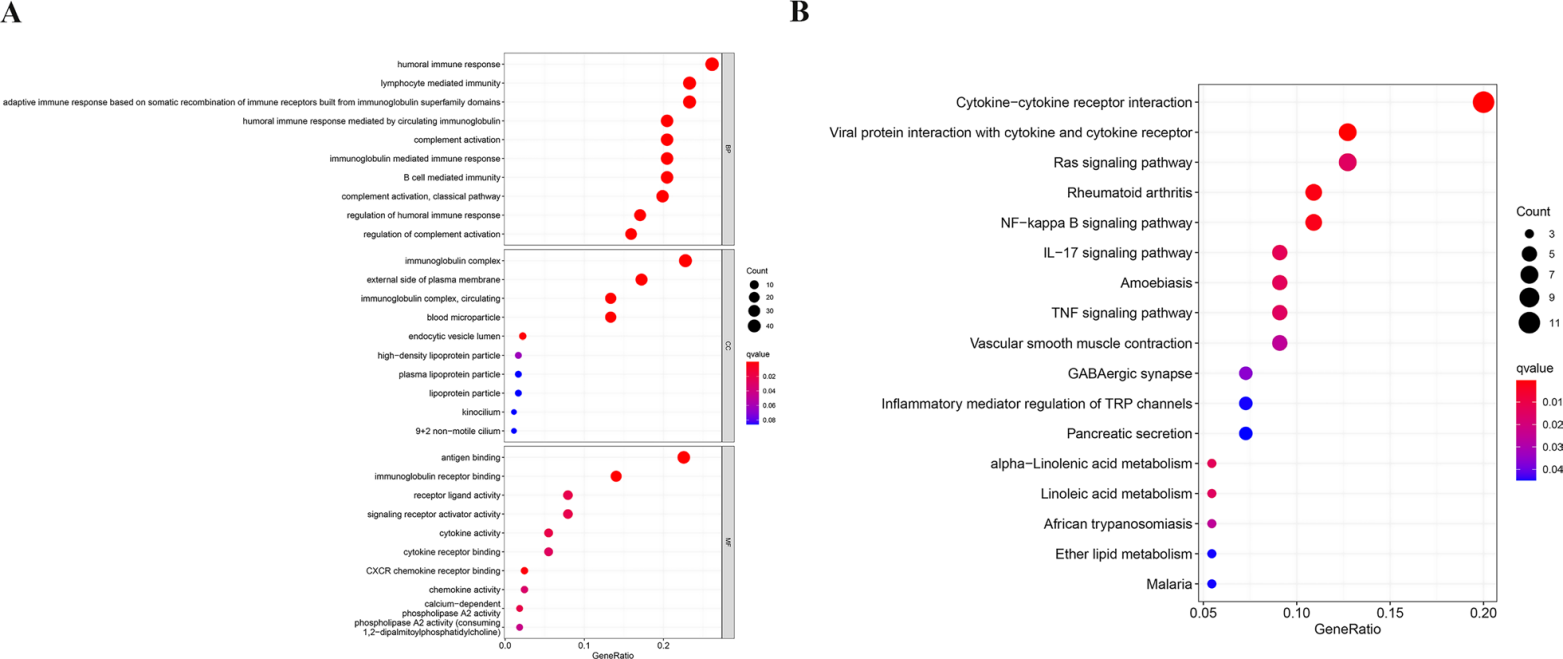
Analyses of functional enrichment. **A** Bubble graph showing GO enrichment. **B** Bubble graph of KEGG enrichment (more dark red indicates more notable differences, and larger bubbles indicate more genes enriched; MF, molecular function; CC, cellular component; BP, biological process; q-value: adjusted p value)

Determining the BPs related to DEGs in the model is of great importance. The “limma” R package was utilized to obtain differentially expressed genes: FDR < 0.01, |log2FC|≥ 1.5. As a result, 381 DEGs in the cohort from TCGA were selected. A total of 378 of the 381 genes in the high-risk group were overexpressed, whereas reduced expression was indicated for the remaining 3 genes (Additional file 4: Table S3). KEGG pathway analysis and GO enrichment analysis of the selected DEGs were conducted (Fig. 5). Of interest, the most enriched biological processes are closely related to immune responses, inflammatory cell chemotaxis, and chemokine-mediated signalling pathways, including B-cell-regulated immunity, humoral immune responses, CXCR chemokine receptor binding, and TNF/NF-kappa B/IL-17 pathways. These findings suggest that the model of prognostic risk based on PRGs is associated with immune responses.

To explore the significance of these six biomarkers in the development of ccRCC, we assessed the underlying biological mechanisms of these hub genes by GSEA. Interestingly, we found that these genes simultaneously play a positive role in multiple identical immune-related signalling pathways, including B-cell and T-cell receptors, chemokines, cytokine‒cytokine receptor interactions, and natural killer cell-mediated cytotoxicity pathways (Additional file 1: Fig. S4). Therefore, we predict that different immune statuses may be responsible for the observed differences in prognosis between clusters.

To further confirm our speculation about the potential BPs of the six biomarkers, coexpression network analysis was performed to identify the functional modules to which the hub genes belong (Additional file 1: Fig. S5). Genes clustered in the identification module may play the same or similar roles [30]. Eigengenes refer to gene expression profiles that include modular genes summarized by the first principal component. The gene significance (GS) and module membership (MM) of these eigengenes are shown in Additional file 5: Table S4. After comparison, we found AIM2 and NOD2 to be simultaneously affiliated with the brown module; the blue module included two hub genes, GSDMB and NLRP1, and IL6 belonged to the red module. Interestingly, all three modules were positively associated with tumorigenesis (Additional file 1: Fig. S5G). Next, we examined the GO enrichment pathways of the significant modules (brown, blue, and red). The gene function of the brown module is closely related to activation of immune pathways, whereas RNA splicing-related pathways are more enriched in the blue module, with acute inflammatory responses significantly enriched in the red module (Additional file 1: Fig. S6).

### Evaluation of immune cell infiltration between subgroups

**Fig. 6:**
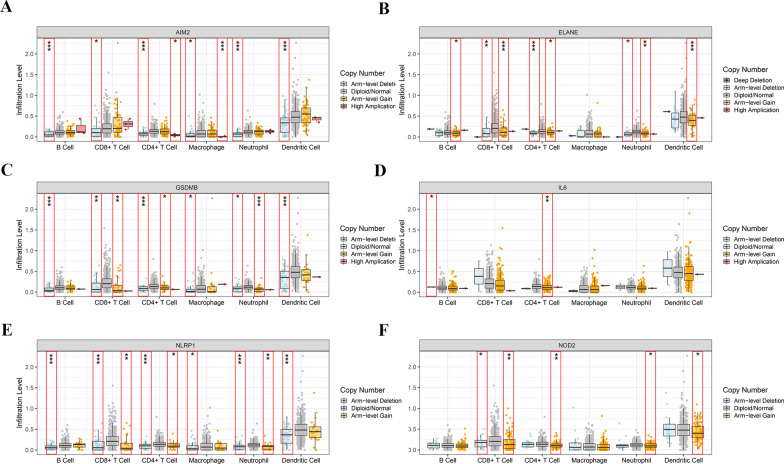
Infiltration levels of immune cells with the six PRG mutants were linked to prognosis. **A** AIM2, **B** ELANE, **C** GSDMB, **D** IL6, **E** NLRP1, **F** NOD2. ***P < 0.001, **P < 0.01, *P < 0.05(navy blue, Deep Deletion; light blue, Arm-level Deletion; grey, Diploid/Normal; yellow, Arm-level Gain; red, High Amplication; P-value refers to the correlation between different mutants and normal group)

**Fig. 7:**
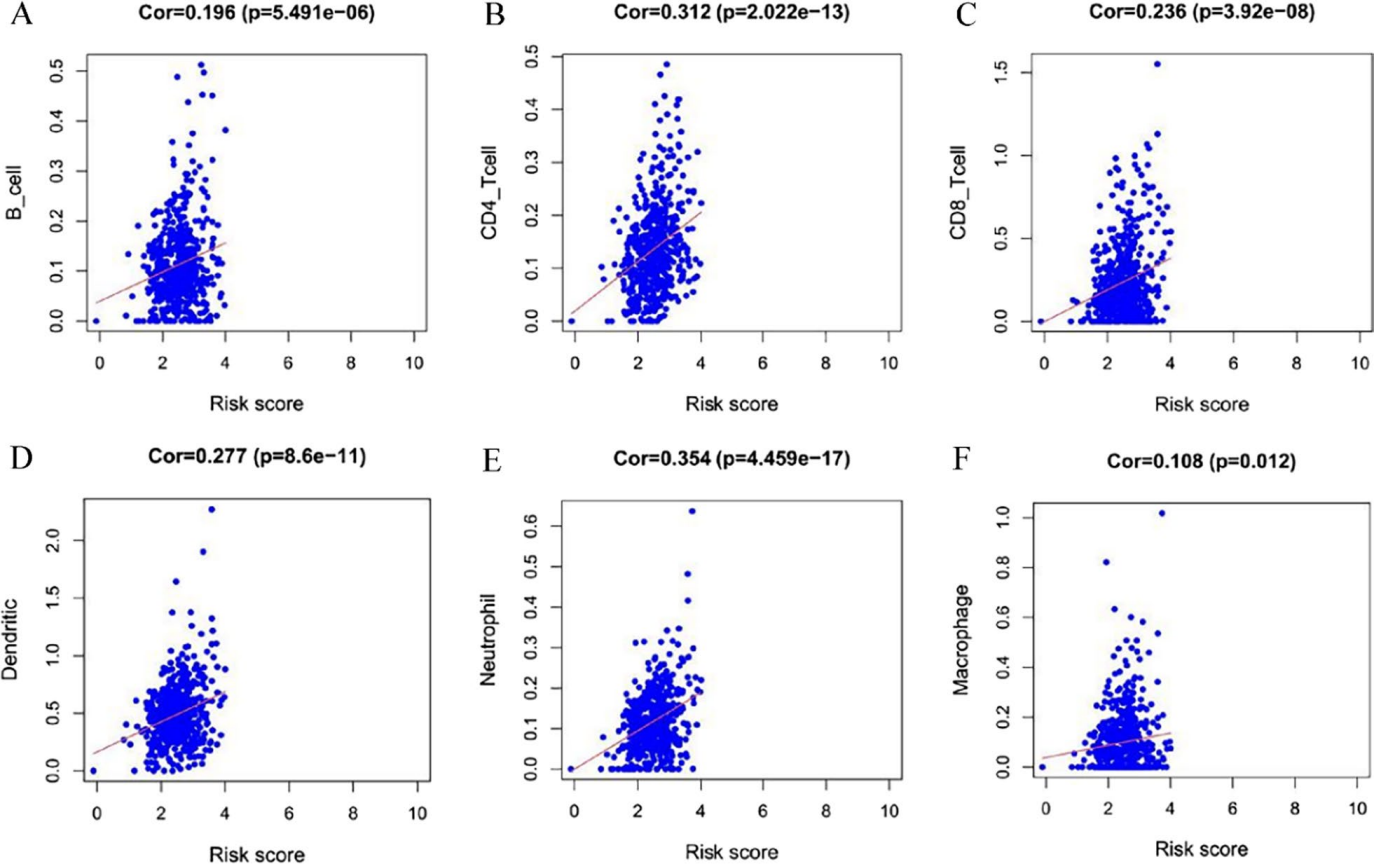
Correlation of risk scores with immune infiltration levels in ccRCC. The risk score of the PRG prognostic model correlated positively with infiltration levels of immune cells .**A** B cells, **B** CD4+ T cells, **C** CD8+ T cells, **D** dendritic cells, **E** neutrophils, **F** macrophages. (Cor > 0, *P* < 0.05)

**Fig. 8:**
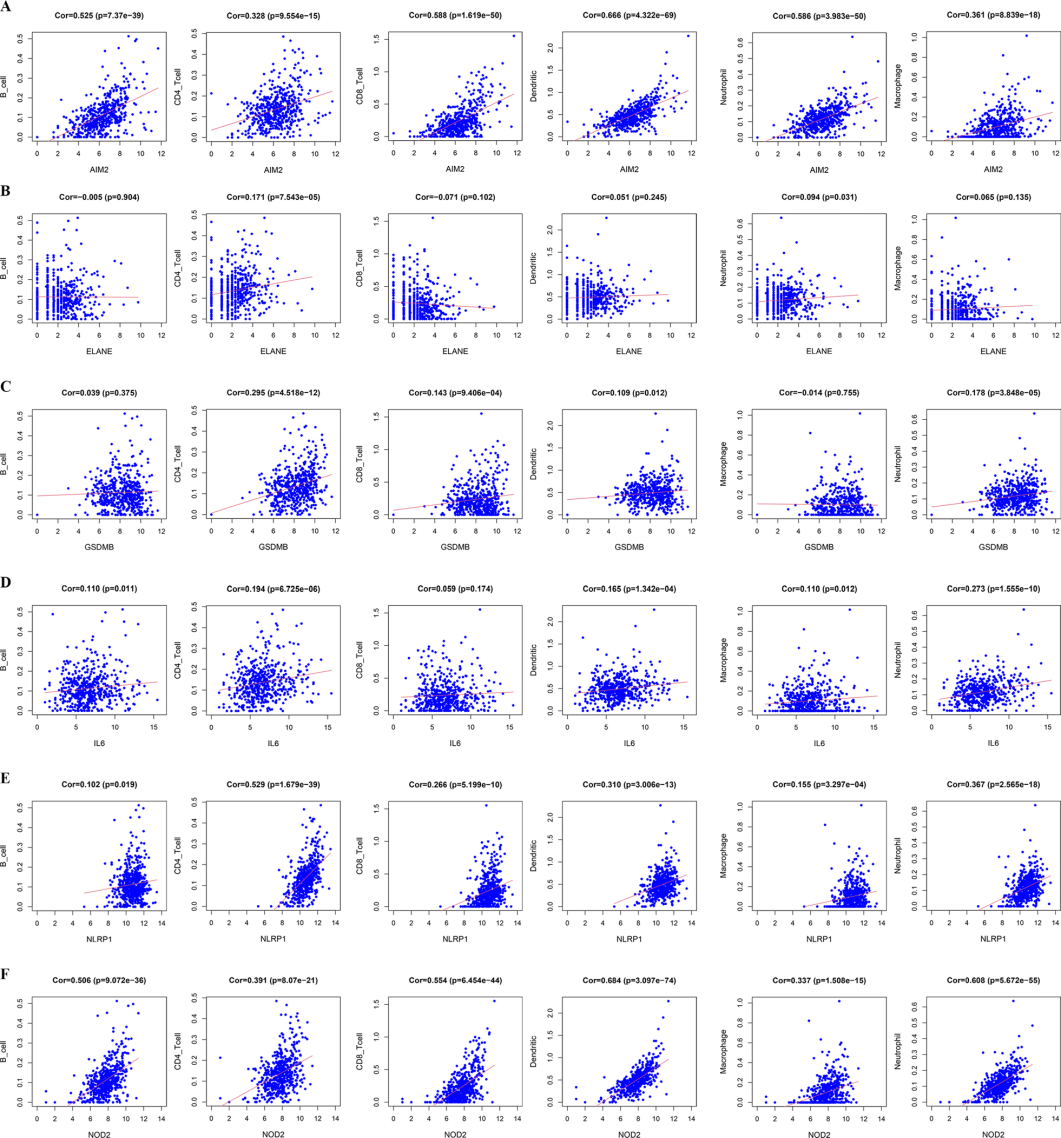
Correlation of six hub genes with immune infiltration levels in ccRCC. (Types of immune cells include CD8+ T cells, B cells, CD4+ T cells, dendritic cells, neutrophils, and macrophages.) |Cor|> 0, *P* < 0.05 was considered significant

According to the above findings, it was proposed that the functions of PRGs in the prediction of ccRCC prognosis might be associated with the immune microenvironment, and the relationship between the infiltration of immune cells and prognosis-related genes was evaluated. Changes in gene copy number may affect the amount of product and thus the traits of the organism, including the content of immune cells in tissues. Consequently, changes in infiltration were explored by using samples with copy number alterations of ELANE, AIM2, GSDMB, IL6, NIRP1, and NOD2. The results showed the copy number of these prognosis-related PRGs to be associated with the immune microenvironment in ccRCC, with AIM2, ELANE, GSDMB, NLRP1, and NOD2 mutations inhibiting infiltration of some kinds of immune cells (Fig. 6A–F). Then, the immune estimation dataset was downloaded from the TIMER database, and correlations between infiltration of 6 types of immune cells (macrophages, dendritic cells, neutrophils, B cells, CD8+ T cells, and CD4+ T cells) and the risk score of the prognostic model were analysed. The data suggest that the risk score of the PRG-related prognostic model correlated positively with immune infiltration (Fig. 7A–F). To define the role played by the 6 prognostic PRGs in immune cell infiltration, correlations with the six immune cells in the TIMER database were investigated separately. As shown in Fig. 8, these six hub genes correlated positively with almost all six types of immune cells.

As a complement, we also selected CIBERSORT for immune microenvironment deconvolution based on bulk RNA-seq. Due to the lack of homogeneity in expression of other immune cells in KIRC samples, we only assessed the association of 6 immune cells with RS. Despite variability in the results obtained by the different computational methods, it is reassuring that T-cell expression exhibited a significant positive correlation with RS in patients with ccRCC (Additional file 1: Fig. S7A). The correlation of six hub genes with 22 types of immune cells was also examined (Additional file 1: Fig. S7B).

**Fig. 9:**
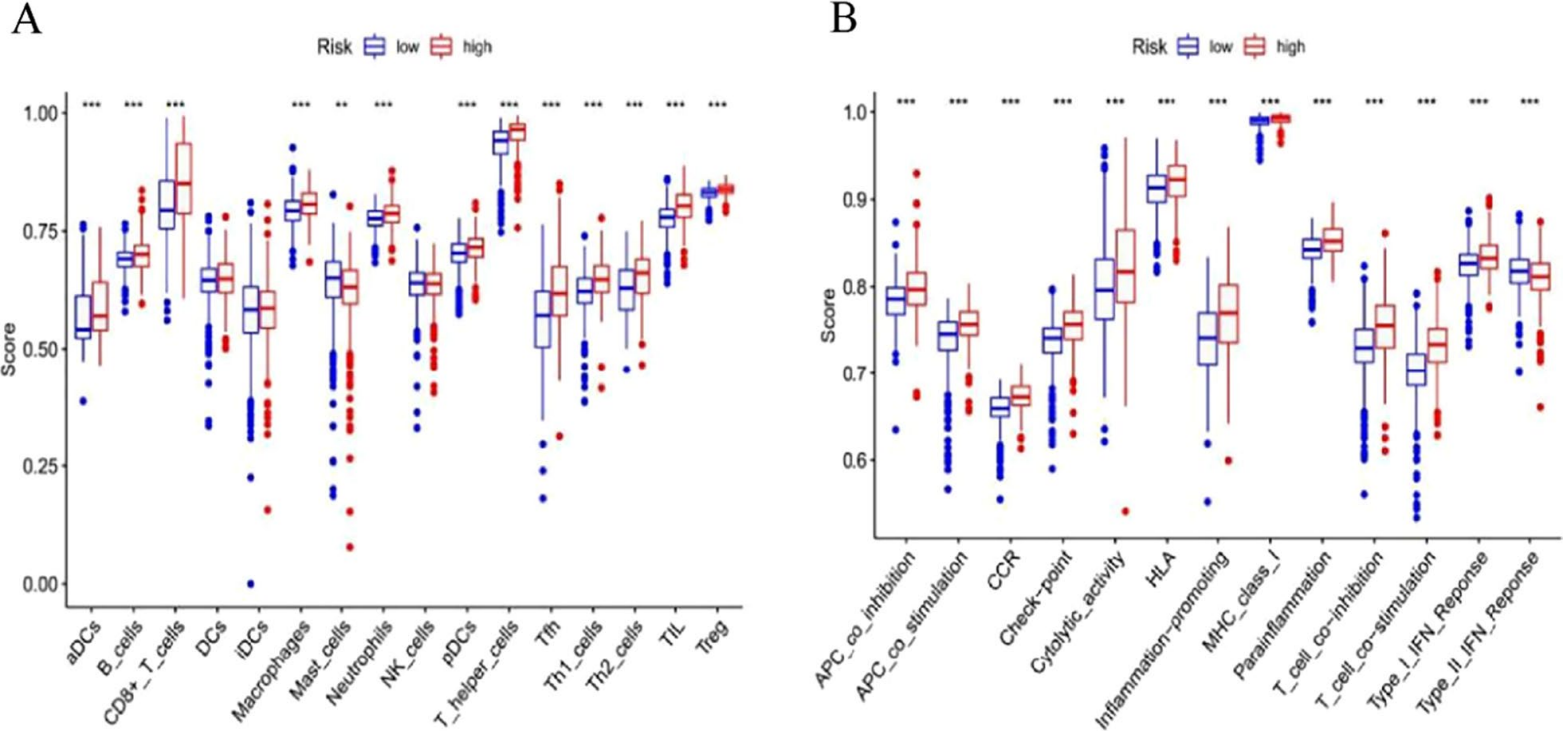
ssGSEA scores concerning immune pathways and cells. **A** Enrichment scores of sixteen types of immune cells between the LR group (blue box) and HR group (red box). **B** Enrichment scores of thirteen pathways linked to immunity between the high-risk (red box) and low-risk (blue box) groups. ***P < 0.001, **P < 0.01, *P < 0.05

The activities of thirteen pathways related to immune responses and enrichment scores of sixteen kinds of immune cells were then compared between the HR group and the LR group of TCGA using ssGSEA (single-sample gene set enrichment analysis). The results revealed increased infiltration in the HR group, especially DCs, Th cells (Th2, Tfh, and Th1 cells), CD8+ T cells, macrophages, B cells, Treg cells, pDCs, TILs (tumour-infiltrating lymphocytes), and neutrophils, compared to the LR group, as shown in Fig. 9A. Figure 9B illustrates that in addition to the type II IFN response pathway, the other 12 immune pathways were more active in the high-risk group than in the low-risk group.

**Fig. 10:**
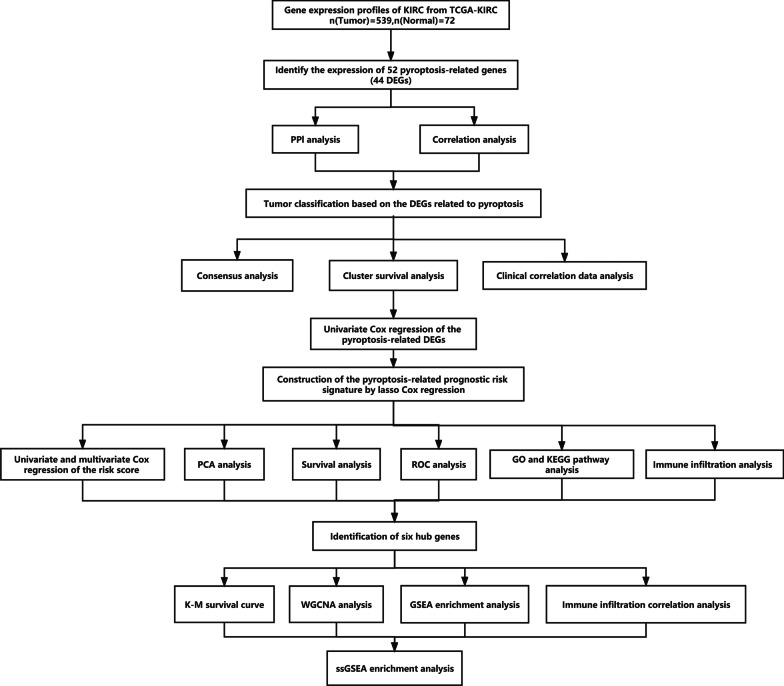
Specific data analysis workflow diagram

The study design and grouping are shown in Fig. 10.

## Discussion

Pyroptosis is a newly recognized kind of programmed cell death that has a dual role in the progression of malignancy and mechanisms of treatment. Inflammatory cytokines are secreted in pyroptosis, and normal cells are stimulated, contributing to progression of cancer [19]. Moreover, pyroptosis can enhance cell death in malignancy, rendering it a possible therapeutic and prognostic target for malignancy [31]. How genes related to pyroptosis interact with each other and whether they have an impact on survival time in ccRCC remain unknown.

In this research, mRNA expression of 52 currently known PRGs was first assessed in normal and ccRCC tissues, and 44 of them were found to be differentially expressed. The 2 clusters in the consensus clustering analysis according to the prognostic PRGs showed significant differences in clinical characteristics, suggesting that pyroptosis in cancer tissues differs in ccRCC, resulting in different OS.

To further evaluate the prognostic value of regulators related to pyroptosis, a 6-gene risk signature was constructed, with different OS in subgroups. Functional analyses showed that the DEGs between the high- and low-risk groups have significant differences in immune-related pathways, consistent with our expectations. Pyroptosis can contribute to accumulation of various inflammatory factors, which also results from activation of inflammasomes [6, 32]. Additionally, cytokine, NF-kappa B, IL-17, Ras, and TNF signalling pathways, which are closely associated with the development of RCC, were enriched.

We also compared pathway activation and immune infiltration between the HR group and the LR group, and the HR group showed increased activities of pathways related to immune responses and elevated levels of IIC in comparison with the LR group. Enhanced infiltration of immune cells was related to poor prognosis, which was consistent with previous studies [33]. Another key result of our research was that the above 6 prognostic genes related to pyroptosis had a significant correlation with immune infiltration, which further suggested the fact that pyroptosis has an essential role in the tumour immune microenvironment.

The current study found a signature of 6 genes linked to pyroptosis (ELANE, AIM2, GSDMB, IL6, NIRP1, and NOD2) and that it plays a role in predicting the OS of ccRCC patients. Decreased expression of AIM2 (absent in melanoma 2) was first found in melanoma [21]. AIM2 is a member of the IFN-inducible PYHIN (pyrin and HIN200 domain-containing) family and acts as a cytoplasmic sensor for DNA that bind to dsDNA (double-stranded DNA) [34]. AIM2 activates caspase-1 via junctional proteins regulated by ASC to facilitate secretion and maturation of IL-18 and IL-1β, thus promoting pyroptosis [35]. Previous research has demonstrated that AIM2 functions as a suppressor in multiple kinds of tumours, such as prostate cancer [36], colon cancer [37], melanoma [38], melanoma [39], and breast cancer but as a promoter in NSCLC (non-small cell lung cancer) [40], OSCC (oral squamous cell carcinoma) [41], and HPV (human papillomavirus)-associated cervical cancer [42]. Therefore, AIM2 might have different effects in different tumours. In this research, expression of AIM2 was significantly increased in tumour tissues in comparison with normal tissues. Furthermore, an increased level of AIM2 expression was closely related to poor survival, and gene mutations in AIM2 might ameliorate infiltration of immune cells. Thus, it has been suggested that AIM2 functions more as a pro-oncogene. The molecular mechanisms of AIM2 in the development of ccRCC currently remain unknown, and our findings for AIM2 might provide new insight into further research.

GSDMB (gasdermin B) was the gene in the prognostic model most linked to the RS, suggesting that GSDMB might be strongly involved in ccRCC. According to previous research on human malignancies, GSDMB is upregulated in tumour tissues, including breast, uterine, gastric, and cervical cancers [43]. It has been demonstrated that GSDMB is located in amplicons and that these genomic regions are frequently amplified in cancer development [44]. Thus, GSDMB might participate in cancer development and metastasis. GSDMB can be cleaved into two fragments by caspase-1. One cleavage form is the N-terminus of the GSDMB protein, which has a molecular weight of 20 kDa. Pyroptosis can be caused by secretion of the N-terminal domain. In contrast, the full-length N-terminal domain and C-terminal fragment do not cause pyroptosis [45, 46]. In general, GSDMB may be a downstream protein of the pyroptosis pathway. The key is whether some factors trigger the upstream mechanism of GSDMB and cause pyroptosis. However, the specific mechanism of GSDMB in ccRCC is not clear. The increased level of GSDMB in ccRCC is related to poor prognosis. This finding may facilitate development of tumour treatment targets.

ELANE (neutrophil elastase gene) is the main serine protease produced by neutrophils and activates proinflammatory cytokines, including IL-1β, IL-18, and TNF-α [47, 48], which are regarded as promoters of pyroptosis. Kambara et al. [49] showed that ELANE cleaves and activates GSDMD and subsequently induces pyroptosis in neutrophils. The ELANE expression level in the HR group was markedly increased compared with that in the LR group, but paradoxically, the neutrophil infiltration score was incredibly higher than that in the LR group. These findings may result from many complex factors driving the difference in gene expression between disease and healthy tissues, particularly levels of genes and PRGs linked to inflammation, such as the proportion of infiltrated immune cells and the differentiation of ccRCC [50, 51]. Such factors might not influence application of the PRG expression signature in diagnosing and predicting ccRCC prognosis. The relationship between expression of pyroptosis genes and infiltration of immune cells, ccRCC differentiation status, and other factors requires further investigation, which may provide new insight for predicting ccRCC diagnosis and prognosis.

NLRP1 (NLR family, pyrin domain containing 1), a bipartite adaptor protein, is considered an apoptosis-associated speck-like protein with an ASC (caspase-recruitment domain). NLRP1 promotes the recruitment process of pro-caspase-1 to the inflammasome complex [52]. GSDMD is cleaved by active caspase-1, allowing the N-terminal domain of GSDMD to form pores in the plasma membrane and triggering the pyroptosis mechanism [10, 15, 53, 54]. NOD2 (nucleotide-binding oligomerization domain-containing protein 2) initiates NF-κB (nuclear factor-κB)-dependent and MAPK (mitogen-activated protein kinase)-dependent gene transcription. In macrophages, NOD2 promotes activation of inflammatory bodies [55]. In our study, NOD2 was highly expressed in the HR group, which might be one of the reasons why we observed higher scores for macrophages in the HR group than in the LR group. At the same time, the activation effect of NOD2 on the NF-κB pathway might have resulted in the high enrichment of this pathway in the KEGG analysis. Interestingly, as one of the genes in the risk prognostic model, the activated NF-kB pathway increases the amount of IL-6 mRNA [56]. In advanced metastatic breast cancer cells, overactivated NF-kB promotes chromatin accessibility at the IL-6 promoter region and enhances transcription of the IL-6 gene [57]. Appropriate expression of IL-6 is of great importance for human immune defence, but its sustained production has a pivotal role in the occurrence of multiple inflammation-related diseases and cancer [58]. In our study, IL-6 was significantly highly expressed in the HR group, and we speculate that NOD2 might act as an upstream initiator of IL-6 to promote IL-6 expression by activating the NF-kB pathway.

The purpose of our research was to categorize cases with ccRCC into different subtypes, screen DEGs, establish a model of prognosis, and connect pyroptosis with prognosis. Although we conducted multiangle and multiomics validation, there are limitations in this study. All analyses were performed using the KIRC cohort from TCGA, and other independent datasets should be examined for validation. Additional in vivo and in vitro experiments are also required to verify our findings. Pyroptosis, particularly the mechanism in ccRCC, has not been sufficiently investigated. In addition, the occurrence of human diseases is caused by combined action of multiple factors, such as the social environment, genetics, and psychology. As tumours develop, multiple cell death modes may coexist and interact [59]. Therefore, a Mendelian randomization study should be carried out to clarify the causal relationship between the six signature genes and prognostic outcomes [60, 61]. To make the results more reliable, effects of other confounding factors also need to be tested. We initially investigated the prognostic value of the 6 genes related to pyroptosis in the prognostic risk model, providing theoretical support for future studies.

## Conclusions

In conclusion, a comprehensive and systematic bioinformatics analysis was performed, and we found a prognostic gene signature related to pyroptosis, including six genes (ELANE, AIM2, GSDMB, IL6, NIRP1, and NOD2), for ccRCC patients. Moreover, the risk score in the prognostic model according to the 6 PRGs, which are associated with the immune microenvironment, was able to act as an independent prognostic factor for ccRCC.

## Supplementary Information

**Additional file 1.** Supplementary figures.

**Additional file 2. Table S1** 52 pyroptosis-related genes.

**Additional file 3. Table S2** Markers of immune cells and pathways.

**Additional file 4. Table S3** DEGs between high- and low-risk groups.

**Additional file 5. Table S4** GS and MM of eigengenes.

**Additional file 6. Table S5** The characteristics of 539 TCGA KIRC patients.

**Additional file 7. Table S6** Different grouping information of 539 TCGA KIRC patients.

## Data Availability

In this study, different web-based datasets were used for data analysis. The web links to all the original data sources are listed below. The sequencing data of transcriptome RNA and the clinical data were downloaded from The Cancer Genome Atlas Program (TCGA) (https://portal.gdc.cancer.gov/) data portal. PPI network analysis was conducted by using the STRING database (https://string-db.org/). The relevant data used in the analysis of immune infiltrating cells were downloaded from the TIMER database (https://cistrome.shinyapps.io/timer). All data generated during the analysis process of this study are available from the corresponding author on reasonable request.
