# Supplementary material for: A pyroptosis-associated signature plays a role in prognosis prediction in clear cell renal cell carcinoma
Source: BMC Med Genomics. 2022 Sep 26;15:204. doi: 10.1186/s12920-022-01339-0 (PMC9513884; doi:10.1186/s12920-022-01339-0)
Supplement: Supplementary file 1 — Additional file 1. Supplementary figures. [file 12920_2022_1339_MOESM1_ESM.docx]

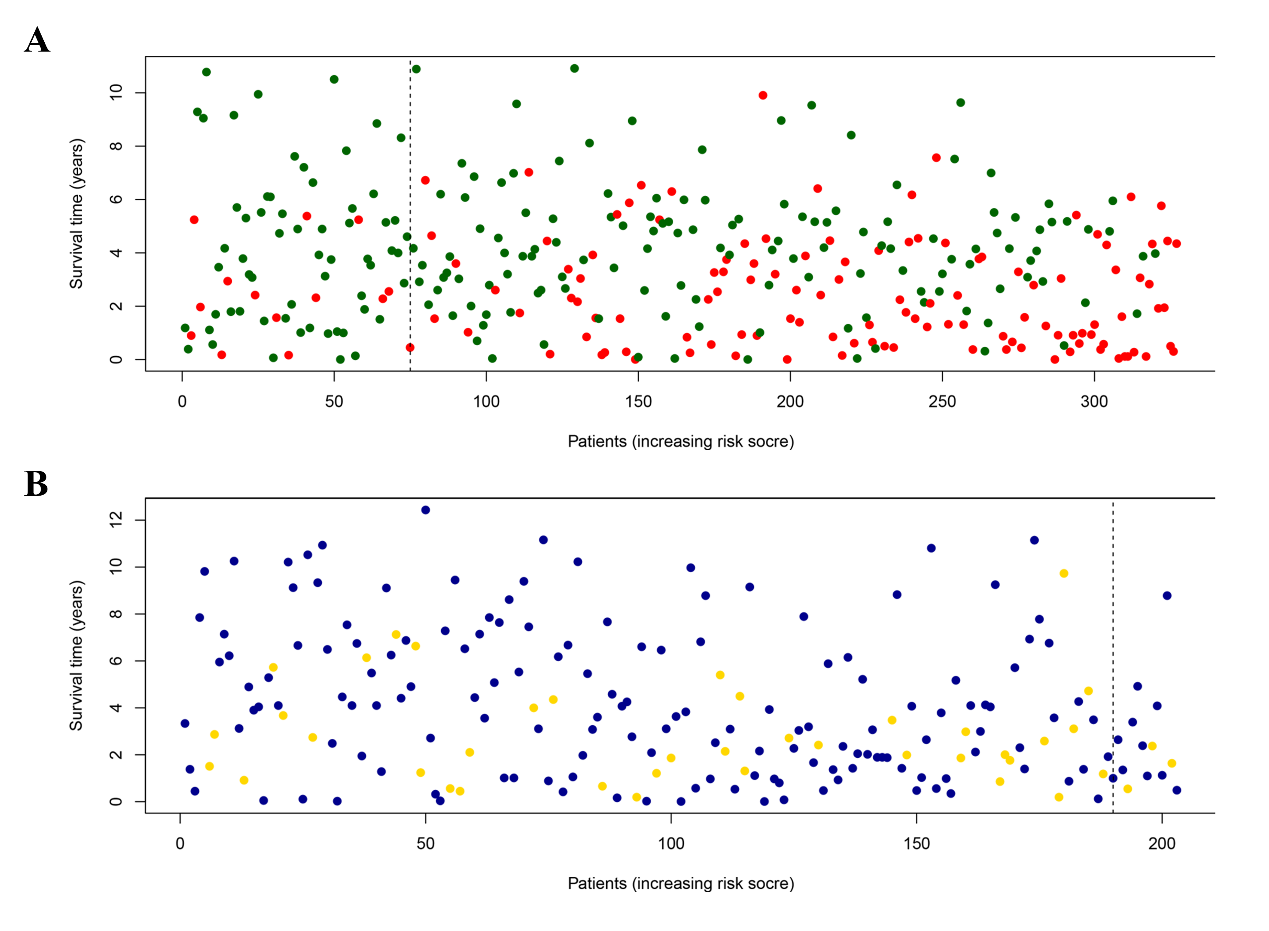


**Fig. 1** The distribution of the risk score in the two clusters. **A** Cluster 1 (high-risk cases, red; low-risk cases, green). **B** Cluster 2 (high-risk cases, yellow; low-risk cases, blue)


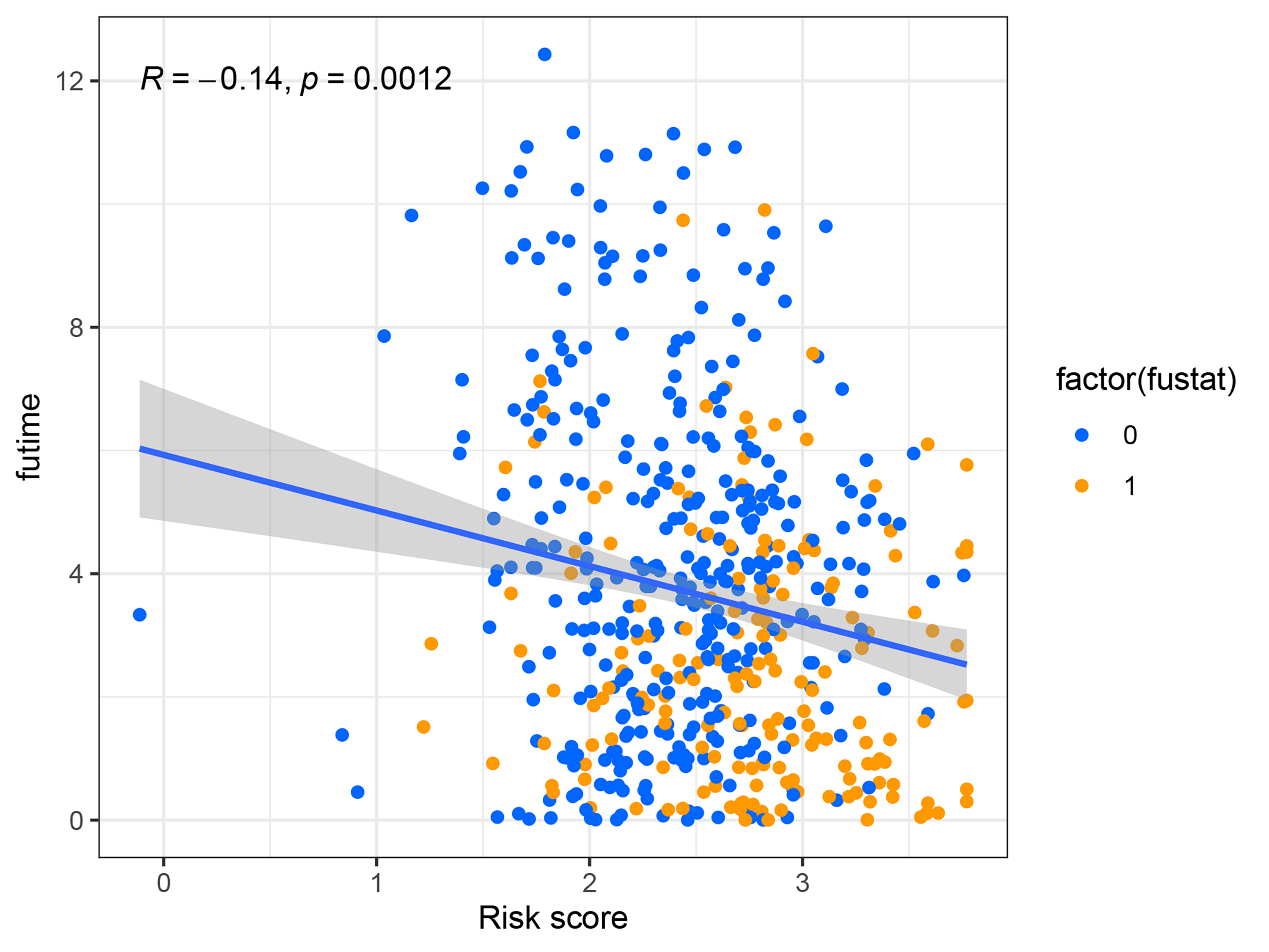


**Fig. 2** Correlation of RS and OS as continuous variables (alive samples, blue; dead samples, orange).


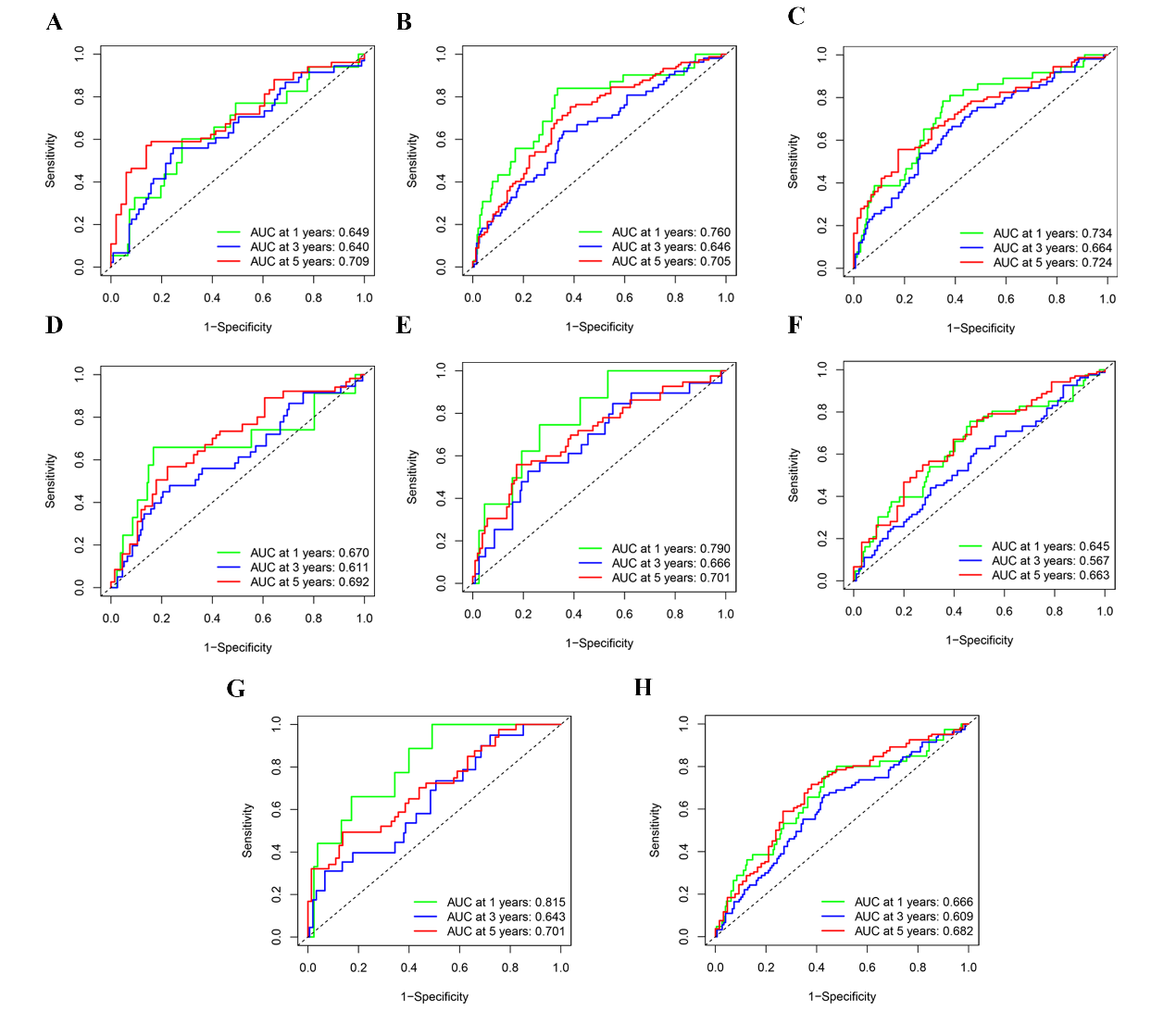


**Fig. 3** Predictive efficiency of the RS indicated by ROC curves in different populations. **A** Female. **B** Male. **C** Age≥60. **D** Age<60. **E** Stage 1-2. **F** Stage 3-4. **G** Grade 1-2. **H** Grade 3-4.


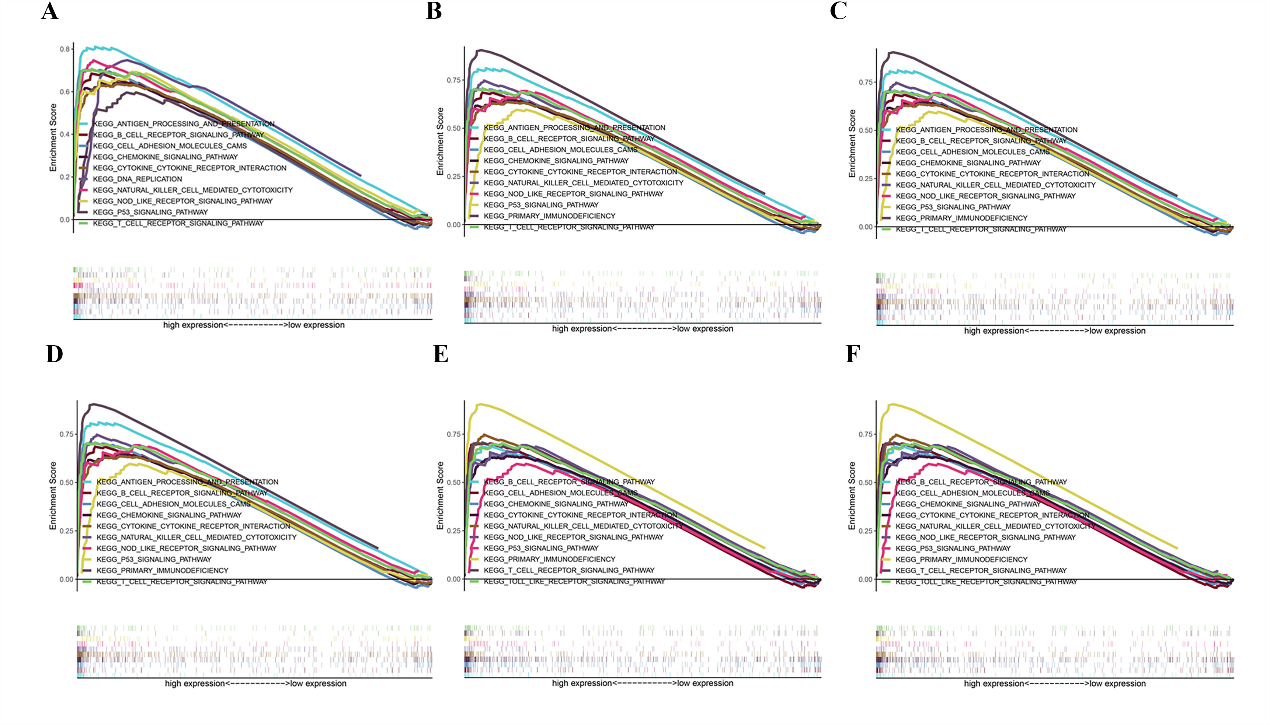


**Fig. 4** GSEA enrichment analysis for six hub genes. **A** AIM2. **B** ELANE. **C** GSDMB. **D** IL6. **E** NLRP1. **F** NOD2.


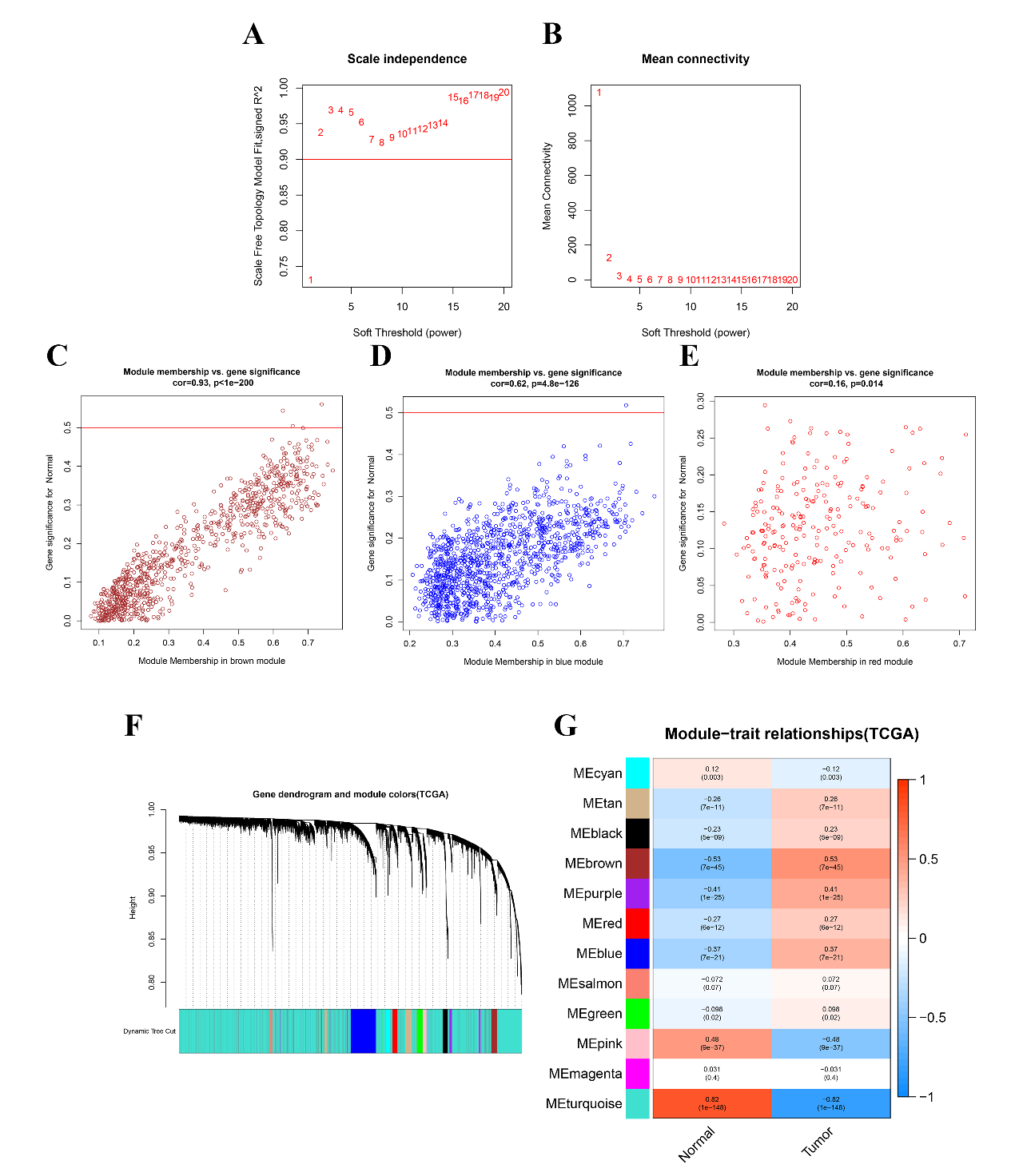


**Fig. 5** WGCNA analysis of DEGs among 539 KIRC samples in TCGA cohort. **A** Determination of soft threshold **B** Mean connectivity corresponding to different soft thresholds. **C** Scatter plotting of the correlation between GS and MS in the brown module. **D** Scatter plotting of the correlation between GS and MS in the blue module. **E** Scatter plotting of the correlation between GS and MS in the red module. **F** Gene hierarchical clustering method based on 1-TOM matrix sorts the clustering dendrogram of co-expression network modules. Colors are assigned to each module by a Dynamic Tree Cut algorithm. **G** Heatmap showing correlations between tumorigenesis and module eigengene values. Blue and red represent negative and positive correlations, respectively. Numbers outside parentheses represent correlations, numbers inside parentheses represent gene significance levels


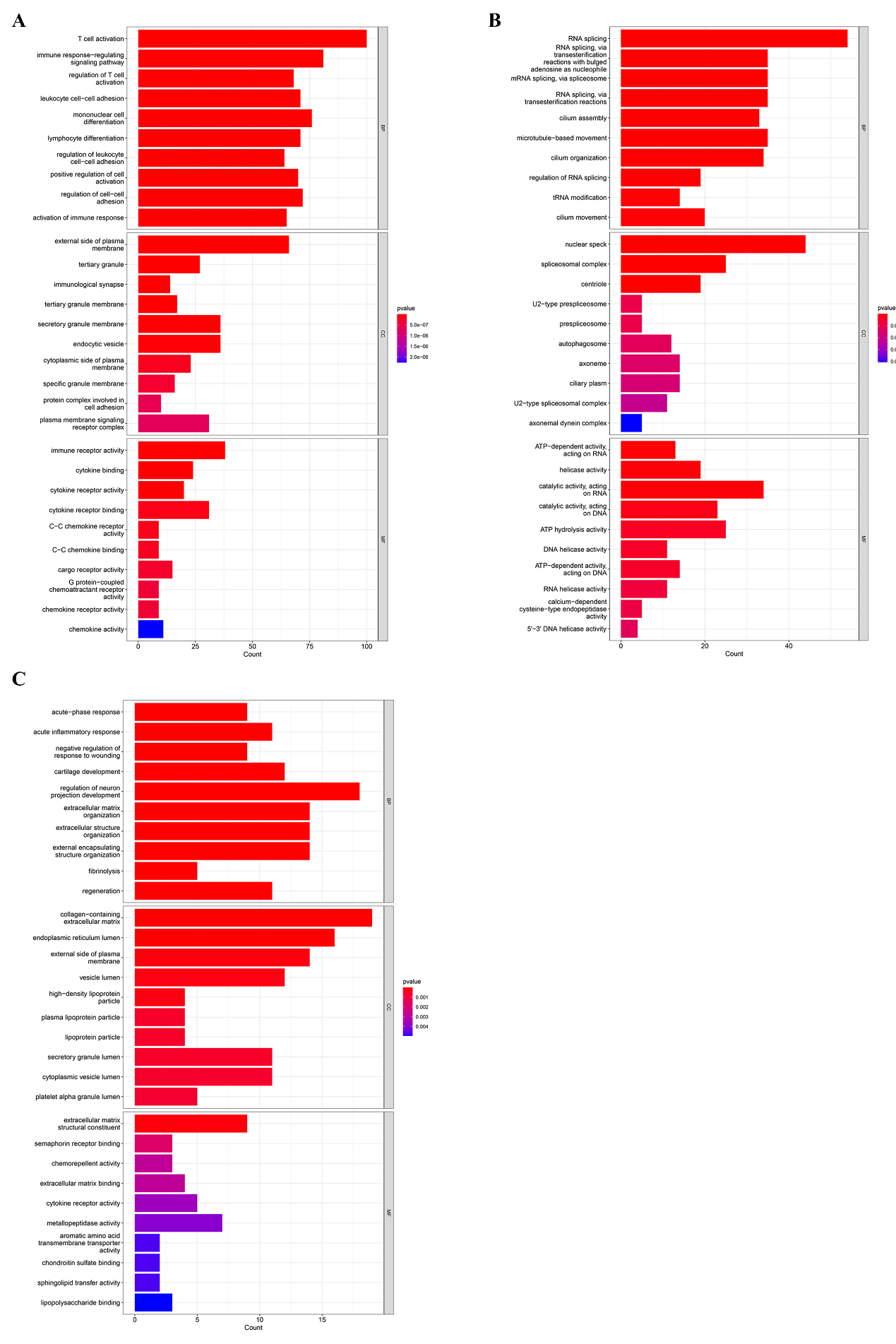


**Fig. 6** GO enrichment analysis of significant modules. **A** Brown model **B** Blue model. **C** Ren model (more dark red indicates more noticeable differences; MF, molecular function; CC, cellular component; BP, biological process).


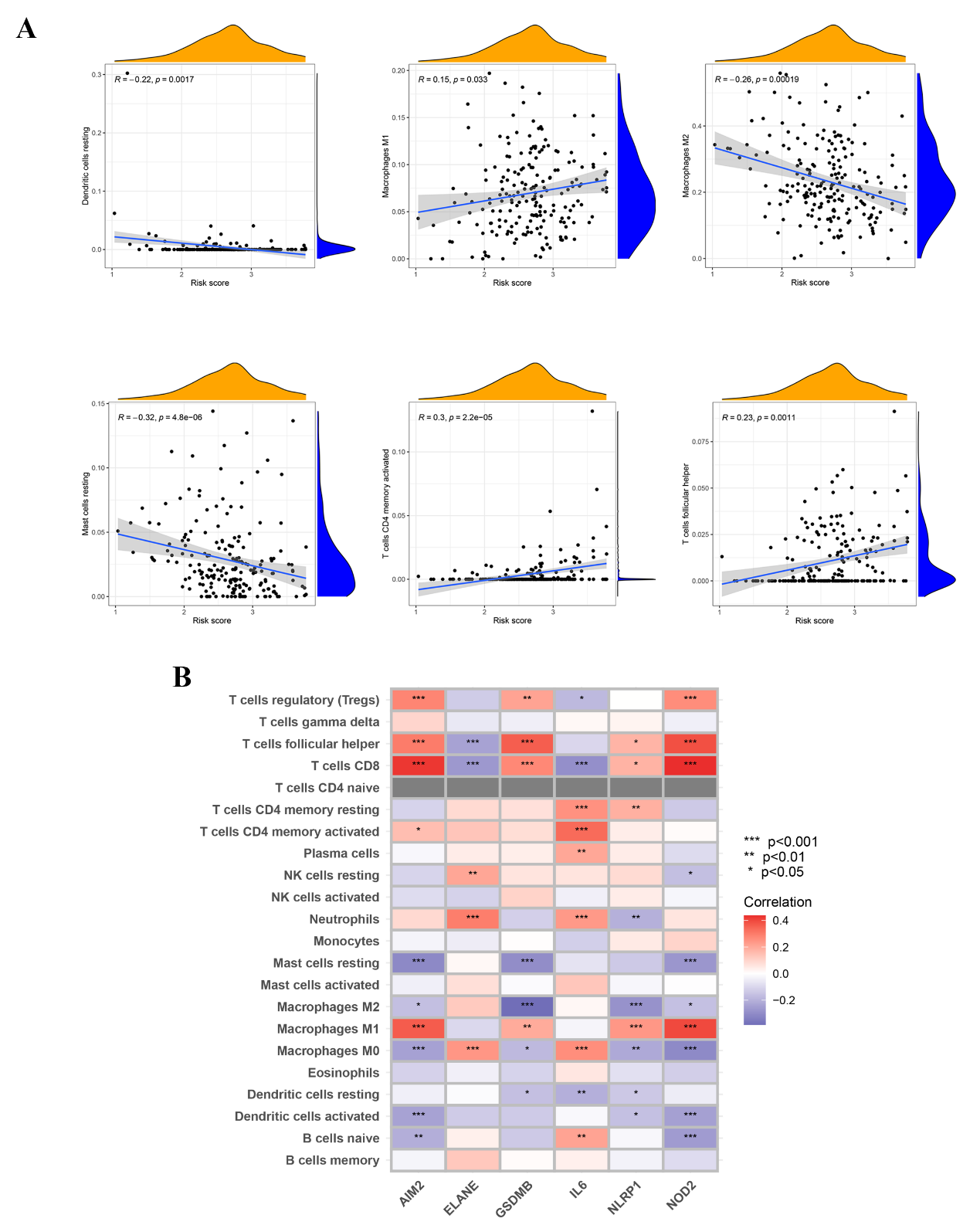


**Fig. 7** Correlations of immune infiltrating cells with RS and hub genes in ccRCC. **A** Scatter plot of the correlation between immune infiltrating cells and risk score. **B** Heatmap of the correlation of the six hub genes and 22 immune infiltrating cells (The expression of T cells CD4 naive in KIRC samples could not be estimated. more dark red indicates a more significant correlation, ***p < 0.001, **p < 0.01, *p <0.05).
